# Progress in Pediatrics

**Published:** 1901-12-21

**Authors:** 


### Progress in Pediatrics.

**Acquired Aortic Disease in Childhood.**—Acquired aortic lesions are admittedly rare in childhood, and are, according to Dr. Marfan,[1] of two types, the rheumatic, and the atheromatous. In the former he places four varieties. First, there is pure aortic insufficiency, revealed by the same physical signs as in adults, but producing little disturbance to the patient. Secondly, there is aortic insufficiency associated with aortitis. The systolic murmur in this condition is often ascribed to stenosis of the aortic valves, but is really due to a roughening of the valves and the aorta in their neighbourhood. The inflammatory process may extend further along the aorta and even be accompanied by cylindrical enlargement of that vessel, as shown by an increased area of aortic dulness on percussion. Serious symptoms accompany this condition such as asthmatic seizures, suffocating attacks with vomiting, and a fatal termination may come on suddenly. A third variety is sometimes met with in which mitral lesions are present in addition to the preceding, while in a fourth variety pericardial adhesions may be present. On the subject of the atheromatous type of aortic disease, Dr. Marfan says this is extremely rare, but its existence has been proved at various necropsies. The cause of the condition is quite unknown but it is not rheumatism. The clinical sign of most importance is a systolic murmur at the base from chronic aortitis, and not from aortic stenosis, as is usually supposed. Few symptoms are present until adult life is reached when compensation breaks down. The lines of treatment indicated by Dr. Marfan are as follows:—In rheumatic cases treatment by salicylate of soda is to be pushed vigorously. In cases with good compensation he advises the avoidance of mental or physical fatigue, of heavy meals, of the drinking of much fluid, and of digitalis, while a prolonged residence at the seaside or in the hills, combined with the use of opium and bromides will delay any temporary disturbance, should such be present. In the forms with cardiac weakness and pericardial adhesions he uses digitalis freely.

**Fœtal or Congenital Rickets.**—Under this title many cases have been published, as Dr. Ashby[2] points out, which were not congenital rickets but were examples of achondroplasia, an entirely different affection. Other cases present themselves which cannot be thus classified, and of which the underlying pathological condition is still unknown. As an illustration he relates the case of an infant, seen when a fortnight old, and suffering from spontaneous fractures of the humeri, radius, ulna, and femur. The cranial bones showed imperfect ossification, and the ribs were abnormally soft and bent inwards laterally, as in rickets. The infant was the fourteenth child in the family, and the mother's age was 46. Under treatment the patient made a good recovery, and when seen again at the age of nine months presented no signs of rickets. In discussing this case Dr. Ashby says that osteomalacia, fragilitas ossium, and syphilis may be excluded. The age of the mother and the debilitating effects of numerous pregnancies were probably factors in producing the bony changes, even although these were not accompanied by general malnutrition. In the absence of a pathological examination of the bones, it is impossible to say if real rhachitic changes were present.

**Sudden Death in Infancy and Childhood.**—This subject has an important medico-legal bearing as is shown by the frequent verdict in a coroner's court of death from "over-lying." In the absence of a careful autopsy such a verdict is valueless, and may throw unmerited blame on innocent people. Even with a most careful examination, however, there are numerous cases of sudden death in which no definite cause can be found. The chances of sudden death in an infant under two years of age are much greater than those of an older child. This, according to Dr. A. . Vipond,[3] is due to two factors, first the greater irritability of the nervous system in an infant, and secondly the proneness to acute pulmonary attacks. The nervous instability is manifested by convulsions, secondary to stomach or intestinal irritation, rickets, onset of acute exanthemata, etc., and death is due to asphyxia or heart failure. Death from pneumonia following exposure will be accompanied by changes in the lungs very similar to those of death from overlying or other form of asphyxia. The lungs may be full of froth, and show subpleural hæmorrhages, but in the former case if a section of lung tissue is squeezed between the fingers, muco-pus will exude from the divided ends of the bronchi. Affections of the larynx and trachea, which may be classified as extra-mural, mural, and intra-mural are responsible for many sudden deaths. The most important of the extra-mural causes is the presence of an enlarged thymus gland, which may lead to death from pressure on the vagus or trachea. In the case of wasted infants, as, for instance, from diarrhœa, the possibility of sudden death must always be kept in mind.

**Mental Fatigue in School Children.**—An interesting investigation has been carried out by Dr. Joseph Bellei[4] as to the relations between the daily instruction given in the schools and the mental fatigue that is thereby caused. He used six passages for dictation, of about equal difficulty, and gave the first to the children at 9 A.M., when school commenced; the second at 10 A.M., the end of the first lesson hour; the third at 11 A.M., the end of the second lesson hour; the fourth at 11.45 A.M., immediately before the luncheon hour and midday rest; the fifth at 12.45 P.M., when school was resumed for the afternoon, and the sixth at 2 P.M., during the last half hour of lessons. He then examined the results as to the number of mistakes in these exercises at the different hours. From these he concludes that the morning lessons do not produce great mental fatigue, that the midday rest is of great use, and has not the disadvantage of a long rest in inducing a state of inattention on resuming work, and that, although immediately after the midday rest the mental condition is active, an hour or so of application is sufficient to produce such a mental fatigue as to lead at the end of the afternoon lesson to the worst work of the day. The morning's work consumes the mental energy of the children without fatigue, but afternoon work in addition cannot be long carried on without inducing this condition.

**Raw Milk in Infantile Atrophy.**—In the estimation of many, sterilised milk holds a very high place as a diet both in health and in disease. Escherich maintains that by sterilisation the essential difference between cow's milk and human milk is done away with, and others consider it so digestible that dilution is quite unnecessary even for newly-born infants. On the other hand it is maintained by the opponents of sterilised milk that it is so digestible that it passes out of the stomach before it has been thoroughly acted on by the gastric juice, and consequently it is not so assimilable and the infant's nutrition suffers. Dr. S. Monrad[5] has recorded five cases in which sterilised milk had no nutritive value, and was not digested or absorbed at all, but the children throve well when put on a raw milk diet. These were all cases of infantile atrophy. In explanation of this occurrence he states that undoubtedly the chemical properties of milk are affected by a prolonged high temperature, which induces a lessened coagulability in the casein, a change in the milk sugar, and large fat globules in place of a fine emulsion. He further points out that by common consent the prescribed period of sterilising by Hoxhlet's process has been reduced from 45 minutes to 20, or even 10. Dr. Monrad thinks that raw milk is not to be given to healthy infants as a routine diet, nor in cases of acute gastro-enteritis, but on the other hand when other measures have failed it promises well in cases of infantile atrophy or chronic gastro-intestinal catarrh.

[1] La Semaine Med., March 27, 1901. [2] Lancet, Aug. 17, 1901, p. 441. [3] Montreal Med. Jour., 1901, Vol. XXX., p. 23, and Med. Chron., June, 1901. [4] Lancet, May 11, 1901. [5] Hospitals tidende (Copenhagen), February, 1901, and Med. Chron., June, 1901. [6] Lancet, 1901, Vol. I., p. 1859.

